# Optimal Energy Transfer in Light-Harvesting Systems

**DOI:** 10.3390/molecules200815224

**Published:** 2015-08-20

**Authors:** Lipeng Chen, Prathamesh Shenai, Fulu Zheng, Alejandro Somoza, Yang Zhao

**Affiliations:** Division of Materials Science, Nanyang Technological University, 50 Nanyang Avenue, Singapore 639798, Singapore; E-Mails: CHEN0846@e.ntu.edu.sg (L.C.); pmshenai@ntu.edu.sg (P.S.); M130048@e.ntu.edu.sg (F.Z.); SOMO0001@e.ntu.edu.sg (A.S.)

**Keywords:** optimal energy transfer, natural and artificial light harvesting systems, quantum coherence, non-photochemical quenching, charge separation

## Abstract

Photosynthesis is one of the most essential biological processes in which specialized pigment-protein complexes absorb solar photons, and with a remarkably high efficiency, guide the photo-induced excitation energy toward the reaction center to subsequently trigger its conversion to chemical energy. In this work, we review the principles of optimal energy transfer in various natural and artificial light harvesting systems. We begin by presenting the guiding principles for optimizing the energy transfer efficiency in systems connected to dissipative environments, with particular attention paid to the potential role of quantum coherence in light harvesting systems. We will comment briefly on photo-protective mechanisms in natural systems that ensure optimal functionality under varying ambient conditions. For completeness, we will also present an overview of the charge separation and electron transfer pathways in reaction centers. Finally, recent theoretical and experimental progress on excitation energy transfer, charge separation, and charge transport in artificial light harvesting systems is delineated, with organic solar cells taken as prime examples.

## 1. Introduction

The most abundant source of energy available on Earth comes in the form of sunlight. Despite the huge potential it offers to solve the globally augmenting problem of energy crisis, harvesting the solar light energy efficiently and economically has turned out to be among the most challenging problems faced by mankind. A large number of organisms ranging from quite primitive bacteria to high-order green plants have developed a sophisticated way to harness the light energy by converting it to chemical forms via the highly efficient process known as photosynthesis. This intricate process begins with the absorption of photons by specialized pigment-protein complexes that function as antennae for incident energy. The excitation energy is subsequently transferred along a complicated network of such light-harvesting complexes to special sites called the reaction centers (RCs), where charge separation takes place thereby driving a chain of electron transfer reactions resulting ultimately in the synthesis of adenosine triphosphate (ATP) and nicotinamide adenine dinucleotide phosphate (NADPH) [1]. Among the many astonishing features of photosynthesis are its remarkable efficiency and adaptability. At low light intensities, the fact that almost every captured photon reaches the RCs triggering the charge separation process emphasizes the near 100% energy transfer efficiency. On the other hand, at high intensities, the photosynthetic machinery is protected by a key regulatory mechanism known as non-photochemical quenching (NPQ) which serves to provide a pathway to harmlessly dissipate the excess energy as heat and at the same time maintain an optimal photosynthetic yield [2]. When the trigger for this mechanism to kick in subsides, *i.e*., ambient light intensity returns to normal, the original energy conversion functionality of the photosynthetic apparatus gets restored quickly [2]. Experimental and theoretical research so far has unveiled many intriguing aspects of NPQ, yet, a unanimous consensus on its molecular mechanisms is far from being achieved [3].

The mechanisms that bestow such near-perfect efficiency to natural systems even under widely varying conditions are not fully understood; however, recent advances tantalizingly point to the possible manifestations of quantum effects playing a crucial role. The advent of two-dimensional (2D) Fourier transfer electronic spectroscopy [4,5] has paved the way to directly study energy transfer in photosynthetic light harvesting complexes [6,7,8,9]. The application of this technique to the Fenna-Matthews-Olson (FMO) pigment-protein complex in *Chlorobium tepidum* [7] appear to yield a direct evidence of quantum coherence through the observation of quantum beats signaling light-induced oscillations in both donors and acceptors. The quantum beating lasts remarkably for at least 660 fs, contrasting with the general notion that the electronic coherence decays on the 10–100 fs timescales [10,11]. This wavelike characteristic of the energy transfer rather than incoherent hopping might allow simultaneous sampling of all energy transfer pathways as well as identification of the most efficient to funnel solar energy from pigments to RCs. The initial experiments were performed outside the physiological range of temperatures, but recent studies have observed that electronic quantum beating can last up to 300 fs even at physiological temperatures [8,9]. Moreover, the detection of quantum coherence in the most abundant light harvesting complex on Earth called the Light Harvesting Complex II (LHCII) from green plants [12], indicates that the electronic quantum coherence may universally be an integral part of photosynthetic processes. While various theoretical studies provide constructive viewpoints about the potential roles of quantum coherence in photosynthetic excitation energy transfer (EET), the underlying mechanism regarding the observed long lived quantum coherence is still elusive, when put in perspective that the existence of electronic quantum coherence in such warm, wet and noisy biological systems is truly astonishing. Resolving and extracting the underlying mechanisms that enable the functioning of such a painstakingly optimized process in natural systems is thus fundamentally important not only in advancement of the knowledge frontier but also in designing robust artificial photosynthetic devices operating at a high efficiency.

While a complete overview of artificial systems for light-harvesting is beyond the scope of the present review, a note on organic Photovoltaic (OPV) devices is in order in the present context. OPV devices utilize conductive organic polymers or small organic molecules to convert solar energy into electrical energy by the photovoltaic effect. The light harvesting in OPV devices begins with the absorption of a photon at the donor layer leading to the formation of an electron-hole pair bound together by electrostatic interactions, *i.e*., excitons. The excitons then migrate to a charge separation interface and are split into free holes and electrons by effective fields that are set up in the heterojunction, from which they are transported to respective electrodes. Among the many challenges the OPV technology faces is enabling the transport of excitons to the charge separation interface before their decay occurs. Traditional beliefs that this excitonic transfer occurs via random hopping along the organic molecular chain have been challenged by recent observations of long-lived intrachain electronic and vibrational coherences in resonant energy transfer along systems such as conjugated polymer chain (poly[2-methoxy-5-(2${}^{\prime }$-ethyl-hexoxy)-1,4-phenylenevinylene], MEH-PPV) [13]. The mechanisms of quantum phenomena in the energy transfer between the donor-acceptor molecules in a functional OPV cell still remain unclear, and their clear elucidation carries a great potential in forming guidelines on constructing OPV devices with much more efficient energy transfer properties by optimal device design.

In an energy transfer process that entails a highly complicated interplay between quantum coherence, trapping and dissipation [14,15,16,17,18,19,20], identifying the optimal conditions for attaining the best quantum efficiency is challenging evident from the huge body of literature devoted to it. In this review, we will restrict ourselves to draw a broad overview on the exploration of the optimal energy transfer mechanisms in photosynthetic light harvesting systems. We will particularly emphasize on potential implications of the design principles of natural light harvesting systems in optimizing the operation and performance of artificial devices. This review is structured as follows. In the next Section II, we will present an overview of the studies focused on efficiency and optimization of excitonic energy transfer (EET) in various natural light harvesting systems under different physical conditions. Section III reviews the findings on the energy and charge transport in artificial light harvesting systems with special attention given to organic molecular crystals. Outlook is presented in Section IV.

## 2. Efficient Energy Transfer in Natural Light-Harvesting Systems

### 2.1. State of the Art Theoretical Treatment of Energy Transfer in Light Harvesting Systems

Despite the greatly complicated structure of the photosynthetic machinery in various light-harvesting organisms, specialized light harvesting complexes are well known to harvest and deliver the excitation energy to the photochemical reaction centers with remarkable efficiency [1]. The ultra-efficient energy transfer in light harvesting systems has inspired generations of researchers attempting to unravel its mechanisms and replicate them in artificial systems harnessing solar energy. Recent experiments provide encouraging signs that the quantum coherence may prove to be the key to optimize transfer pathways and maximize efficiency [7,8,9]. These experimental results have reinvigorated the relentless pursuit of intriguing questions as to how nature attains such a great efficiency in highly noisy biological systems and how to understand the optimization conditions in terms of optimal design theory. Recent reviews on the existing state affairs in photosynthesis research can be found in [21,22,23].

In the past decades, tremendous efforts have been devoted to investigate energy transfer in light harvesting systems theoretically. A common assumption undertaken in photosynthesis studies is that energy dynamics remain in the one-exciton manifold, *i.e*., at every time there is only one exciton in the system, in accordance to the rather large (∼ ns) exciton lifetime in Bchl aggregates [24]. Accordingly, only the first molecular excited state is considered (for example the $Q_{y}$ transition in Bacteriochlorophyll species) and an arrangement of *N* chromophores is modelled as an ensemble of two-level systems. This approach is generally correct, although two exciton states need to be included to simulate ESA (Excited State Absorption) pathways in 2D spectroscopic signals. Finally, a general Hamiltonian of an exciton system ($H_{\mathrm{ex}}$) coupled ($H_{\mathrm{ex}-\mathrm{bath}}$) to a bath of oscillators ($H_{\mathrm{bath}}$) is formulated:

(1) $$\begin{array}{ccc} H & = & H_{\mathrm{ex}}+H_{\mathrm{bath}}+H_{\mathrm{ex}-\mathrm{bath}}\\ & = & \sum_{n}\epsilon_{n}a_{n}^{†}a_{n}+\sum_{n\neq m}J_{nm}a_{n}^{†}a_{m}+\sum_{q}\omega_{q}\left(b_{q}^{†}b_{q}+1/2\right)\\ & & +\sum_{nq}g_{nq}\hbar \omega_{q}\left(b_{q}+b_{q}^{†}\right)a_{n}^{†}a_{n} \end{array}$$

where $a_{n}^{†}\left(a_{n}\right)$ is the creation (annihilation) operator of an excitation (*i.e*., an exciton or a charge carrier) with on-site energy $\epsilon_{n}$, and $b_{q}^{†}\left(b_{q}\right)$ is the creation (annihilation) operator of a phonon with frequency $\omega_{q}$ and a wave-vector *q*. $J_{nm}$ is the electronic transfer integral coupling two molecules *n* and *m*. The electron-phonon coupling is usually diagonal in the site basis and it provides relaxation among excitonic states. Off-diagonal coupling is often included in the context of organic semiconductors (see below).

Highly accurate and efficient methods have been developed and applied to investigate the energy transfer and optical properties of the light harvesting complexes which are often described by an electronic system coupled to its thermal environment. Many light harvesting systems are in the intermediate coupling regimes in which the electronic coupling between chromophores and the coupling between the chromophores and the environment are comparable, making the traditional Förster and Redfield theories based on second order perturbation with respect to the electronic coupling and excitation-environment interaction inappropriate for treating EET dynamics. Furthermore, the characteristic timescales of the environmental reorganization and system dynamics are often of similar magnitude, leading to the invalidity of Markovian approximation. From a theoretical point of view, dynamical description in such intermediate regimes are challenging due to the intrinsic non-perturbative and non-Markovian nature of the system. The hierarchical equation of motion (HEOM) approach [25], a non-perturbative, non-Markovian open quantum system method, has been widely used to study the excitation energy transfer in light harvesting systems. For example, the long-lived electronic coherence observed in the FMO complexes is successfully reproduced by this method [26]. To improve the computing efficiency of HEOM algorithm, Strümpfer *et al.* have employed parallel computers for the investigations of EET in large light harvesting systems: single light-harvesting complex 2 (LH2) from purple bacteria [27,28], LH2-LH2 [27,28], LH1-LH2 [29] and LH1-RC systems [30]. By taking advantage of the high performance of Graphic Processor Units (GPU), Kreisbeck and coworkers have implemented the HEOM approach on GPU (GPU-HEOM) [31] and studied EET in the FMO complex [31,32] and the LHCII [33]. Alternatively, a scaled algorithm for hierarchical equations of motion has been developed [34] to dramatically reduce the number of auxiliary density matricies used, and is applied to study the energy transfer dynamics in LH2 complexes [35,36] and the two-dimensional electronic spectroscopy (2DES) of FMO complexes [37].

**Figure 1:**
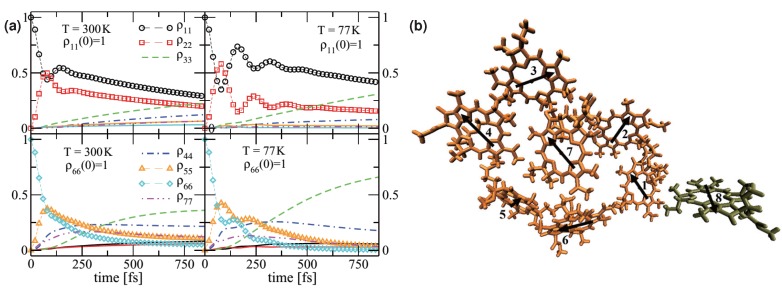
(**a**) Time-dependent occupation probabilities of all seven Fenna-Matthews-Olson (FMO) sites produced by quasiadiabatic propagator path-integral (QUAPI) method [45] for T = 300 and 77K with $\rho_{11}$(0) = 1 and $\rho_{66}$(0) = 1 for the measured FMO spectrum [19]; (**b**) The bacteriochlorophylls (BChls) a in an FMO monomer where the eighth BChl a is nearby the neighboring monomer. The arrows in panel (**b**) are the directions of transition dipole moments [52].

It is noted that the standard HEOM method involves the Lorentz decomposition of the spectral density, which can be done systematically based on certain sum-over-pole schemes. Yan and co-workers dramatically increased the efficiency of HEOM by applying the Padé spectrum decomposition techniques [38,39,40]. Moreover, the optimal HEOM construction with accuracy control has been achieved for any bath of Brownian oscillators [41]. In addition, different HEOM+stochastic implementations have been proposed, among which Shao *et al.* developed a HEOM scheme by decoupling the interactions between the system and its heat bath via the Hubbard-Stratonovich transformation and interpreting the influence functional as a stochastic field induced by the environment [42,43]. A hybrid stochastic hierarchical equation of motion (sHEOM) approach was proposed [44] to decrease the temperature dependence of the performance of the ordinary HEOM algorithm by treating the real part of the bath correlation function with stochastic unraveling. The iterative real-time quasiadiabatic propagator path-integral (QUAPI) approach provides another numerical exact method to study open quantum systems for any form of spectral densities. Nalbach and coworkers [45,46] applied this method to study energy transfer in the FMO complex. They reproduced the same coherent dynamics calculated by HEOM as shown in Figure 1 under different initial conditions and temperatures.

In order to overcome the computational bottleneck of above numerical exact methods, Zhao *et al.* developed a set of efficient trial wave functions, the Davydov *Ansatz*, by using Dirac-Frenkel time-dependent variational principle, and have applied it to investigate the energy transfer in various light-harvesting complexes [47,48,49,50,51]. The energy transfer pathways in dual-LH2 systems are systematically investigated by the Davydov D${}_{1}$ *Ansatz* [47,48], it is found that the phase of the transmission amplitude through the LH2 complexes is crucial for constructing the coherent excitonic energy transfer. These studies also reveal that symmetry breaking caused by the dimerization of bacteriochlorophylls and correlation between two rings may increase the energy transfer efficiency by introducing multiple intra/inter-ring transfer pathways. By incorporating the Davydov *Ansatz* into the nonlinear response function formalism, Zhao *et al.* developed a new theoretical framework to calculate the third-order non-linear signals of molecular aggregates [49,50]. Both singly and doubly excited excitonic states as well as the contributions from stimulated emission, ground state bleach, and excited state absorption can be handled by this theory in a unified way. The femtosecond double pump single molecule signals of molecular aggregates can be also calculated within this theoretical framework [51].

Most recently, Zhao and coworkers have employed this theoretical framework to study exciton transport in the chlorosome complex. In the chlorosome antenna complex of green bacteria, hundreds of thousands of bacteriochlorophyll pigments are tightly coupled to each other via dipole-dipole interaction. It is mostly found in the phylum Chlorobi (green sulfur bacteria) [53] and Chloroflexi (filamentous anoxygenic phototrophs) [54]. The chlorosome complex exhibits some unique characteristics which are not shared by common photosynthetic systems. In the first place, it is a self-aggregated structure, constituted mainly by Bacteriochlorophyll c (Bchl c) and lesser contributions of carotenoids and quinones. Secondly, unlike any other photosystems, the interior part of the chlorosome is entirely constituted by aggregated chromophores and does not present any protein scaffold within it. The assembled chromophores are in contact with the baseplate, an additional two-dimensional structure embedded in the chlorosome’s membrane which is mainly constituted of dimerized Bchl a-CsmA pigment-protein units [55]. The chlorosome constitutes the largest known photosystem, presenting an ellipsoidal shape whose dimensions are around 150 nm × 50 nm × 25 nm. The detailed structure of the chlorosome is still unclear due to the inability to obtain precise crystallographic data because of its highly disordered configuration. Nevertheless, it is agreed that chromophores in the interior of the chlorosome are assembled in two-dimensional networks of curved lamellae [56], and rod structures [57,58] connected to a surrounding baseplate. Ganapathy *et al*. [57] proposed syn-anti Bchl c monomers as the building blocks of the chlorosome’s arrangement, although others [59] have proposed dimer-based units.

Although the optical properties [60,61,62,63], and isolated exciton dynamics [64,65,66,67,68] of the chlorosome complex have received much attention, there is still no comprehensive study of polaron dynamics in the chlorosome which describes accurately the detailed interaction between the exciton manifold and lattice phonon vibrations. Raman spectroscopy [69,70] performed on the chlorosome has unveiled a series of intense low frequency peaks (100–200 ${\mathrm{cm}}^{-1}$) in addition to the characteristic high frequency intramolecular modes of bacteriochlorophyll pigments that may originate from vibronic coherences.

**Figure 2:**
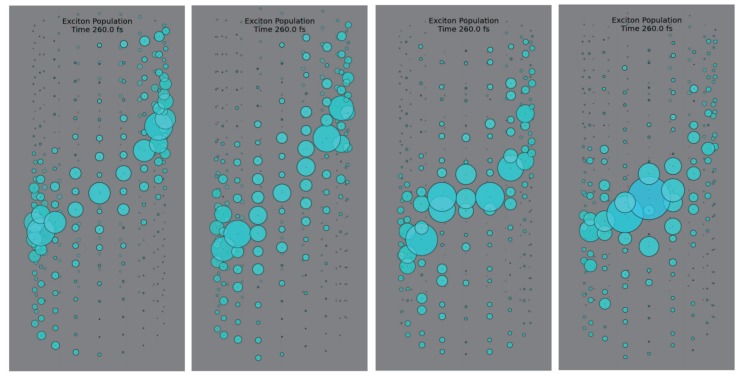
Snapshot of exciton dynamics at 260 fs in the presence of dynamic disorder for a fully localized initial state in a single site belonging to the center of the rod. Periodic boundary conditions have been applied. Several structural models are considered. From left to right: no distance dimerization nor vertical shift, shift, dimerization, shift and dimerization. Superdiffusive behaviour is found in any case during the first 400 fs (no static disorder included).

**Figure 3:**
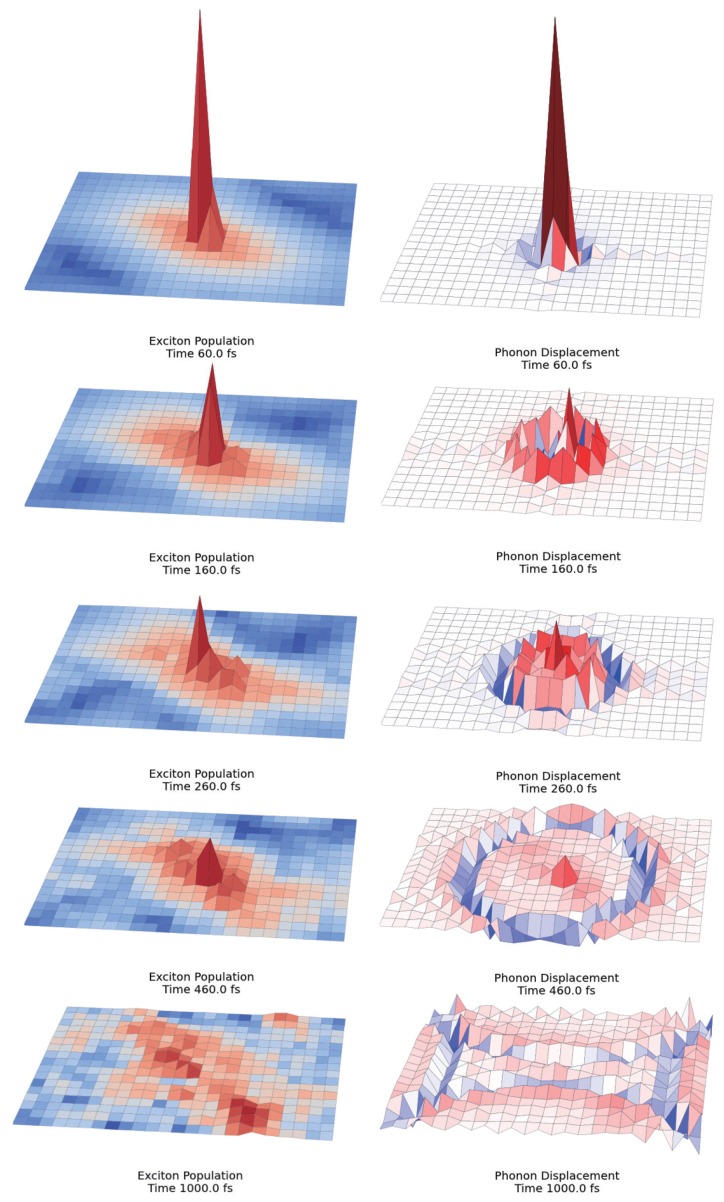
Polaron dynamics in a 18 × 25 lattice. Exciton populations are shown at left and phonon displacements at right. Interchromophoric couplings have been calculated according to the dimer-shift model of rod aggregates for 25 rings, 18 sites per ring. Logarithmic normalization has been applied to the colormap of exciton dynamics to stress differences among sites. Nevertheless, total diffusion on the same complex occurs in less than 400 fs. The rod structure has been folded into a plane for easier illustration

The large scale structure of the chlorosome antenna presents a great challenge for the simulation of exciton dynamics in the presence of dynamic disorder. Notwithstanding, by taking advantage of the parallel structure of GPU, it is now possible to solve the dynamics for this polaron *ansatz* encompassing thousands of interacting chromophores in a dephasing environment. In the case of modelling the acoustic phonon vibrations, the system contains *N* sites and *N* phonon modes. Therefore, the total number of variational parameters is $N^{2}+N$ with *N* complex numbers for the exciton amplitude at every site and $N^{2}$ complex numbers for the phonon displacement of every mode at every site. Regarding the computation time of this algorithm the simulation of 1 ps dynamics for 360 sites, 360 lattice modes (129,960 degrees of freedom) is accomplished in just 5.5 h (including energy dynamics, coherence size and error analysis calculations). The time scaling of the algorithm is found to grow slightly worse than linearly (computation time $∼N_{\mathrm{dof}}^{1.2}$ being $N_{\mathrm{dof}}$ the total number of degrees of freedom). This is possible by virtue of the highly parallel GPU implementation. Alternatively, in the case of very large systems, the total number of degrees of freedom may be significantly reduced by retaining only a reasonable number M<<N of phonon modes. This is the case when only a few phonon modes are strongly coupled to the system or precise knowledge of the environment’s spectral density is available. In that case the total number of variational variables is N+N×M, which may be dramatically lower than $N^{2}+N$. Figure 2 shows the snapshot of exciton dynamics at 260 fs in a 18×25 lattice with interchromophoric couplings extracted from a model inspired by experimental characterization of the chlorosome. Due to the lack of consensus regarding the precise arrangement of chromophores in the chlorosome, several structural motifs (Figure 2) such as distance dimerization along the rod axis and vertical shifts providing helical pathways are analysed independently and their optical and diffusion properties are compared to each other. Super-diffusive behavior is found in every case during the first 400 fs, although the addition of static disorder will severely decrease the extent of this regime. The corresponding exciton populations as well as phonon displacement for dimer shift model of rod aggregates with 25 rings and 18 sites per ring are shown in Figure 3.

In addition to the Davydov variational method, various approaches, e.g., the multichromophoric Förster theory [71,72,73,74], stochastic Schrodinger equation [75] and coherent Modified Redfield theory [76,77], have also been applied to investigate the EET in photosynthetic systems as well as the method developed by Mancǎl and coworkers for the weakly coupled chromophore aggregates [78]. A stochastic process which accounted for the difference between correlation functions for the quantum and classical bath was also employed by Zhu *et al*. [79] into the Zusman and quantum Fokker-Planck equations and this approach produced the exact quantum dissipative dynamics as HEOM. An extensive analysis of the LH2 combining molecular dynamics, quantum chemistry and a polaron model was performed by Damjanović *et al.* [80].

### 2.2. Quantum Efficiency and Average Trapping Time

**Figure 4:**
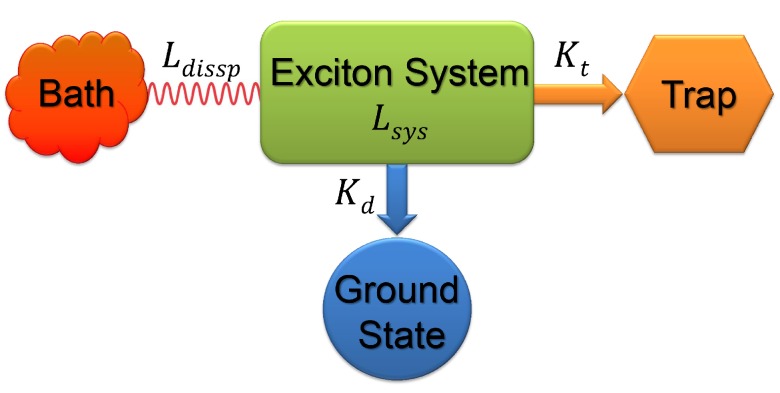
Illustration of an excitonic system coupled to a dissipative environment and subjected to decay to the ground state and trapping at the charge separation state [14].

Coherent and incoherent hopping dynamics have been treated by kinetic mapping of quantum dynamics that includes high-order corrections [14]. It was found that the dissipative effects of the environment can be optimized to yield a maximal efficiency, and the modulation of the efficiency arises from the interference between energy transfer pathways and exists in networks that form at least one closed loop. A simple model for describing the excitonic dynamics in typical natural light harvesting systems is shown schematically in Figure 4. The exciton generated upon illumination, which is treated as the system, is dissipated by the coupling to the environment and may face non-radiative decay to ground state apart from its desired fate of utilization by the reaction center, referred to as the trap. Hence, the exciton dynamics for the light harvesting system follows the Liouville equation [14,81,82],

(2) $$\dot{\rho }\left(t\right)=-L\rho \left(t\right)=-\left[L_{\mathrm{sys}}+L_{\mathrm{dissp}}+L_{\mathrm{decay}}+L_{\mathrm{trap}}\right]\rho \left(t\right)$$

where ρ is the reduced density matrix of the excitonic system, and each of the Liouville superoperators *ℒ* characterizes a distinct dynamic process. The evolution operator of the purely excitonic system $L_{\mathrm{sys}}$ that is completely coherent can be written as $L_{\mathrm{sys}}\rho =i\left[H,\rho \right]/\hbar$, where ${\left[H\right]}_{nm}=\left(1-\delta_{nm}\right)J_{nm}+\delta_{nm}\epsilon_{n}$ with $J_{nm}=J_{mn}^{*}$ being the strength of the transition dipole-dipole interaction between two distinct sites and *ϵ* being the site energy. The Liouville superoperators corresponding to the two irreversible energy loss processes that originate from the decay of the exciton to the ground state and the trapping of the exciton at the charge-separation site can be expressed as: ${\left[L_{\mathrm{decay}}\right]}_{mm,nn}=\left(k_{d,m}+k_{d,n}\right)/2$, and ${\left[L_{\mathrm{trap}}\right]}_{mm,nn}=\left(k_{t,m}+k_{t,n}\right)/2$, where $k_{d,n}$ and $k_{t,n}$ are phenomenological decay and trapping rates at site *n*, respectively. The system-bath interaction, $H_{SB}=\sum_{m}\left|m\rangle \langle m\right|B_{m}$, is utilized to describe the exciton dissipation dynamics, where $B_{m}$ is the linear quantum operator of the bath [81,82,83].The bath induced fluctuations of excitation transition are determined by the time-correlation function, $C_{mn}\left(t\right)=\langle B_{m}\left(t\right)B_{n}\rangle$, which is related to the spectral density $J_{mn}\left(\omega \right)$ by $C_{mn}\left(t\right)=\int_{0}^{\infty }\left[\coth\left(\hbar \beta \omega /2\right)\cos\left(\omega t\right)-i\sin\left(\omega t\right)\right]J_{mn}\left(\omega \right)d\omega$. In the infinite temperature limit, Haken *et al.* showed that the classical white noise follows $\langle \delta \epsilon_{m}\left(t\right)\rangle =0$ and $\langle \delta \epsilon_{m}\left(t\right)\delta \epsilon_{n}\rangle =\Gamma^{*}\delta \left(t\right)\delta_{m,n}$, where $\delta \epsilon_{m}\left(t\right)$ is the site energy fluctuation and $\Gamma^{*}$ is the pure dephasing rate [84]. Under this approximation, the coupling to the environment may be conveniently described by the Bloch-Redfield equation, ${\left[L_{\mathrm{dissp}}\right]}_{nn,mm}=\left(1-\delta_{nm}\right)\Gamma_{nm}^{*}$. At finite temperature, however, theoretical treatment of quantum dissipative dynamics must take into account the detailed balance and the memory effect associated with slow bath relaxation.

The energy trapped at the reaction center and the decay of exciton to the ground state described by $L_{\mathrm{trap}}$ and $L_{\mathrm{decay}}$ represent two possible channels for irreversible exciton energy loss with the former being the effective mode [85,86]. The efficiency of energy transfer is gauged by the quantum yield *q*, also known as, the trapping probability [14,87,88],

(3) $$q=\frac{\sum_{n}k_{t,n}\tau_{n}}{\sum_{n}k_{t,n}\tau_{n}+\sum_{n}k_{d,n}\tau_{n}}$$

In the equation above, $\tau_{n}$ is the mean residence time at each site of the exciton system defined as the integral over the population, *i.e*., $\tau_{n}=\int_{0}^{\infty }\rho_{n}\left(t\right)dt$, where the population $\rho_{n}$ is the diagonal element of the density matrix $\rho_{n}=\rho_{nn}$. In photosynthetic systems, $k_{t}^{-1}$ and $k_{d}^{-1}$ are typically on the order of ps, and ns, respectively. The trapping rate is thus much larger than the decay rate, thereby implying the near-unity quantum yield. The $k_{d}$ dependence of the residence time then becomes negligible, and the quantum yield can be approximated as,

(4) $$q\approx \frac{1}{1+k_{d}\langle t\rangle }$$

where $\langle t\rangle =\sum_{n}\tau_{n}\left(k_{d}=0\right)$ is the mean first passage time to the trap state without the presence of the constant decay, *i.e*., the average trapping time. Quantum yield and average trapping time have been analyzed in detail in the framework of single molecule statistics [89,90,91,92,93,94,95,96,97,98,99,100,101,102,103,104,105,106,107].

**Figure 5:**
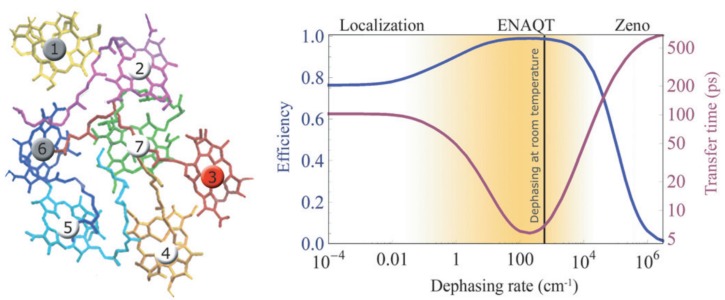
(**Left panel**) Quantum transport network structure of FMO protein. The gray sites and red site represent initial states and final trapping site for the quantum transport network, respectively; (**Right panel**) Efficiency (blue) and transfer time (red) as a function of the pure-dephasing rate for the FMO complex. The vertical line is the estimated optimal dephasing rate at room temperature, and the trapping rate is 1 ps${}^{-1}$ [85].

The most intriguing finding from recent experimental and theoretical investigations is the possible role of environmental noise in assisting quantum transport such that the intricate system-environment interaction may even lead to optimization of energy transfer efficiency (ETE) [7,8,9,85,88,108,109,110,111,112,113,114]. The Haken-Strobl model based study of quantum transport in the FMO complex by Rebentrost *et al.* revealed that ETE can be enhanced by a dynamical interplay of the system with pure dephasing, induced by a fluctuating environment [85]. The left panel in Figure 5 depicts the quantum transport network structure of the pigments in FMO complex, in which pigments 1 and 6 bear the initial state while pigment 3 assumes the role of final trapping site. The right panel in Figure 5 presents the efficiency and transfer time as a function of the pure-dephasing rate. It was argued that the intrinsic static disorder in the system suppresses transport if the noise levels are low. At very high noise levels as well, decoherence effectively produces “watchdog effect” (quantum Zeno effect [115]) that suppresses transport. However, at intermediate noise levels the dephasing noise and quantum coherent effects act harmoniously to achieve an optimal quantum efficiency, manifesting the so-called environment-assisted quantum transport (ENAQT). Although it is only applicable to describe Markovian bath fluctuations, Haken-Strobel model generally captures the dephasing behavior of fluctuating environment in the high temperature limit sufficiently well. In order to circumvent this infinite temperature assumption in the Haken-Strobl model and the failure of Redfield approach in intermediate or highly dissipative regime, Wu *et al.* applied the generalized Bloch-Redfield (GBR) equation to systematically investigate the quantum efficiency of the FMO protein complex and phycocyanin 645 (PC 645) under various physical conditions, including temperature, reorganization energy and spatial-temporal correlations in noise [116]. They found that the maximal ETE is achieved at an intermediate level for various variables. The reorganization energy and the bath relaxation rate (the inversion of bath temporal correlation), in particular, yield a nonmonotonic dependence and thus lead to optimal ETE, albeit only under certain dissipation strength regimes. The HEOM method was also applied to investigate the EET in a dimer system [117] and an optimal energy transfer rate was obtained with medium reorganization energy. Calculated energy transfer rates agree with the results from Redfield equation with extremely small reorganization energy and coincide with the Förster rate in the large reorganization energy region. It is interesting to note that the seven-pigment model structure of the FMO complex employed by the aforementioned studies on the EET efficiency turned out to be inaccurate in the light of the most recent crystallographic analysis. The existence of an eighth chromophore, which is thought to serve as a link between the chlorosomes and remaining seven chromophores was discovered [118,119]. The influence of this additional bacteriochlorophyll (Bchl) on the dynamics and efficiency of FMO was soon incorporated in a study based on GBR and noninteracting blip approximation (NIBA) [120]. The results showed that the population oscillations observed between sites 1 and 2 in the seven site model may be completely suppressed in the eight site model. They attribute the suppression of the population oscillations to the large energy difference between site 8 and the remaining sites. The resulting initial conditions for sites 1 and 2 are effectively incoherent distributions, and it is this dephasing that suppresses the population oscillations.

Despite these advances in understanding of EET, the underlying question of why optimal transport performance can be achieved only at intermediate noise levels remains puzzling. Huelga *et al.* introduced the phonon antenna principle to tackle with this sophisticated problem [121]. This principle states that the optimal scenario for the transition between two exciton states is such that the energy difference between them matches the maximum of the environmental spectral density. Under this condition, the environmental fluctuations being strongest, may facilitate the transitions between the two exciton states most effectively. At low noise levels, the purely coherent dynamics will mark little enhancement in transport. For the strong dephasing noise, on the other hand, formation of the exciton states and thus the phonon antenna effect will be inhibited. As a result, an intermediate regime in which the strengths of intra-system coupling and system-environment coupling are comparable with each other appears to be the optimal regime for efficient transport. Accordingly, the intermediate regime is the typical regime in which photosynthetic EET processes are sustained [122]. It seems that through millennia of evolution, nature has built numerous elegant design principles that facilitate biological systems to operate in an optimal regime in which neither environmental noise nor quantum coherent dynamics clearly dominate so that both contributions do not merely coexist but enter a fruitful interplay.

### 2.3. Quantum Coherence and Its Interplay with Protein Environments

The first experimental observation of long-lived quantum coherence in the FMO complex [6,7] stands as a crucial milestone in the quest of elucidating the near-perfect EET efficiency of natural light-harvesting systems. Engel *et al.* explored EET dynamics in the FMO complex isolated from *Chlorobaculum tepidum* at 77 K by applying the 2D Fourier transform electronic spectroscopy (2DES) [7]. They claimed to have obtained direct evidence for remarkably long-lived quantum coherence among the electronic excited states of multiple pigments in the FMO complex. They also argued that the observed wave-like characteristics of the energy transfer, rather than the incoherent hopping within the photosynthetic complexes, can explain its extreme efficiency, as it allows the sampling of a vast phase space volume in order to find the most efficient path. Panitchayangkoon *et al.* soon performed the 2DES experimental studies on the FMO complex at physiological temperatures and found to a great surprise that quantum coherence could survive in FMO even at 277 K for at least 300 fs [9]. They attributed this long coherence lifetime to correlated motions within the protein matrix surrounding the chromophores. These findings led to a shift in research focus with great attention given to the understanding of underlying mechanisms of long lived quantum coherence and its potential role for the highly efficient energy transfer in various natural light harvesting systems [14,86,111,112,123,124,125,126,127,128].

**Figure 6:**
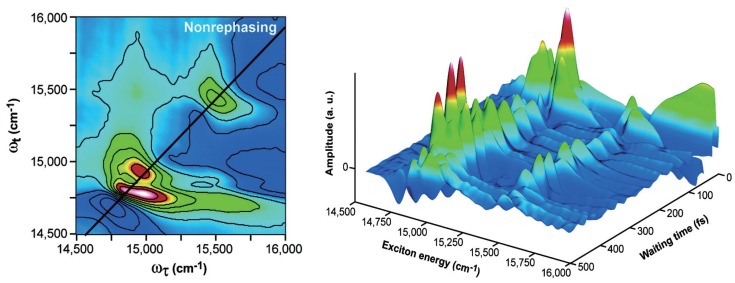
(**Left panel**) The real part of the nonrephasing 2D spectrum of light harvesting complex II (LHCII) at the waiting time 250 fs; (**Right panel**) The amplitude of the diagonal cut of the nonrephasing 2D spectra as a function of waiting time. For both panel, the amplitude increases from purple (negative) to white (positive) [12].

Although most of the recent work is based on the FMO complex as a prototype system to study electronic quantum coherence, researchers have also ventured on the quest to find if such long-lasting quantum coherence is a universal phenomenon. Lee *et al.* carried out two-color photon echo experiments on the RC of purple bacterium *Rhodobacter Sphaeroides* [108], and found dephasing times of 440 and 310 fs at 77 and 180 K, respectively, which are substantially longer than the experimentally estimated excitation energy transfer time scale of about 250 fs [129]. They ascribed the observed long-lasting quantum coherence to the strong correlation between the protein-induced fluctuations in the transition energy of neighboring chromophores, and suggested that correlated protein environments allow the excitation to move coherently in space, thereby enabling highly efficient energy harvesting and trapping in photosynthesis. Calhoun *et al.* applied 2DES to determine the energy landscape in LHCII [12], the most abundant antenna complex in plants containing approximately 50% of the chlorophylls on Earth. LHCII has a trimeric structure in which each monomer contains eight chlorophyll a (Chla) and six chlorophyll b (Chlb) molecules [130]. They explored the 2D nonrephasing spectra by taking advantage of the fact that only the diagonal signals arising from quantum coherence appear in nonrephasing 2D spectra [131]. The left panel of Figure 6 shows the real part of the nonrephasing 2D spectrum at a waiting time 250 fs, and the right panel of Figure 6 depicts the amplitude of the diagonal cross-section of the nonrephasing 2D spectra as a function of waiting time. Quantum beating due to quantum coherence is clearly visible in both Chl-a and -b regimes (corresponding to the low energy and high energy regimes, respectively). By Fourier-transforming the diagonal amplitude of nonrephasing 2D spectra (Figure 6, right panel) along the waiting time axis, they obtained the coherence power spectrum through which the exciton levels can be easily determined.

While above experimental 2D spectroscopy studies reveal the existence of quantum coherence and emphasize its potential role in EET in light harvesting systems, the precise mechanisms regarding the observed long-lived quantum coherence are not yet fully understood from a physical point of view. The electronic coupling between chromophores and the coupling of the electronic transitions to the environment described by the reorganization energy are two interaction mechanisms that determine the dynamics of EET in light harvesting systems [132,133]. In the strong electron-phonon coupling regime, the interchromophore electronic coupling can be treated pertubatively, leading to the Förster theory [134], which describes incoherent hopping of excitation between states localized on single chromophores and completely neglects all coherence between chromophores resulting in coupled rate equations involving populations only. In the opposite limit, *i.e*., weak electron-phonon coupling, it is possible to treat electron-phonon interaction perturbatively, leading to the well-known Redfield theory [135]. The Redfield theory is formulated in the exciton basis, and represents the relaxation of excitons accompanied by coherent evolution of exciton coherences. However, whether or not EET dynamics is quantum coherent is entirely irrelevant to the specific basis we apply to describe the system. The oscillations of off-diagonal elements of density matrix in the exciton basis can be easily transformed into coherent oscillations of the populations in the site basis, which cannot be described by Förster-type theories. Although the Redfield theory has been widely used to study exciton relaxation dynamics in many biological aggregate systems, it is based on the Markovian approximation which assumes instant equilibration of the environment after an electronic transition from the ground to the excited state and on the assumption of weak coupling to the environment for which second order perturbation treatment is valid. While above theories consider only one of the two perturbative limits, there exists an intermediate regime in which the two couplings are comparable. In fact, intermediate regimes are quite common in many light-harvesting systems [122], making perturbative treatment of EET dynamics inappropriate for those systems [132,133]. Furthermore, the characteristic timescales of the environmental reorganization and system dynamics in these systems are often of similar magnitude, so that the Markovian approximation also fails [132,133]. From a theoretical point of view, although exploring physics at such intermediate regimes is challenging due to the intrinsic non-perturbative and non-Markovian characteristics, a number of theories have already been formulated [117,136,137,138,139,140,141,142,143,144].

One of the speculated roles of quantum coherence in EET in natural light-harvesting systems is that it allows the excitation to reversibly sample relaxation rates from all component exciton states and efficiently direct the excitation energy to the lowest energy state [7]. From the perspective of quantum information theory, an interesting suggestion emerged that the system may actually be performing quantum computation for simultaneously searching many states and selecting the optimal path to achieve the high efficiency of the energy transfer. This is a process analogous to Grover’s algorithm [145] which can provide quadratic speed up over a classical algorithm for searching an element in an array of unsorted data [146]. Mohseni *et al.* showed that a purely unitary Grover search algorithm cannot explain the high ETE of the FMO protein [112], and the interplay between quantum coherence and environment-induced noise and decoherence can lead to significantly greater ETE than quantum coherence on its own. However, they suggested that certain nonunitary generalizations of quantum search algorithms could still be developed to describe quantum dynamical systems interacting with a non-Markovian and/or spatially correlated environment. By mapping the EET dynamics to the dynamics of quantum walks, Hoyer *et al.* explored the limits of quantum speedup in the FMO complex [147]. The results showed that there is only short lived quantum speedup (70 fs), as compared to the hundreds of femtoseconds over which the electronic quantum coherence might be able to last. This investigation revealed that the EET dynamics in light-harvesting complexes does not correspond to a quantum search, and the electronic quantum coherence is more likely to contribute to other aspects of transport, such as overall efficiency or robustness instead of yielding dynamical speedup.

**Figure 7:**
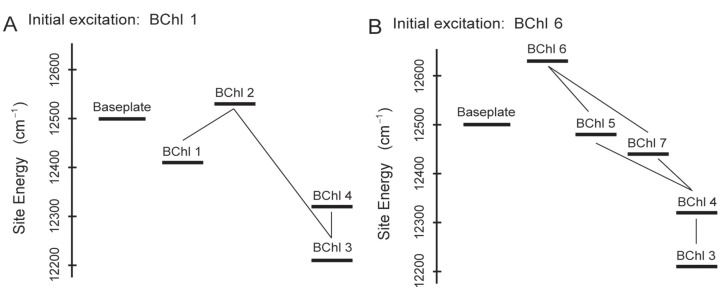
The energy landscapes along the two primary transfer pathways in the FMO complex: baseplate → BChls1 → 2 → 3 → 4 (**A**) and baseplate → Bchls6 → 5,7,4 → 3 (**B**) the relatively strong couplings between BChls are depicted by solid lines [26].

The spatial and temporal dynamics of EET in the FMO complex at physiological temperatures were systematically investigated by Ishizaki and Fleming [26]. Their results revealed that the FMO complex may function as a type of a “rectifier" for the unidirectional energy flow from the chlorosome antennas to the RC by taking advantage of the intricate interplay between quantum coherence and the energy landscape of pigments tuned by the protein matrix. It was proposed that if the EET were to be mainly controlled by diffusive hopping mechanism, trapping in local energetic minima would be inevitable. However, quantum coherence can allow avoidance of the traps to efficiently direct the energy transfer to find the most effective sinks for the excitation (which, in the isolated FMO complex, are linker pigments facing the RC). Energy landscapes along two primary pathways are presented in Figure 7, where Figure 7A shows that if the site energies of the BChls were arranged in monotonic decreasing pattern, the relatively flat energy landscape compared to $k_{B}$T ($k_{B}$ and T are the Boltzmann constant and temperature, respectively) would facilitate backward transfer of excitation away from the RC at physiological temperatures. Due to the large energy gap between BChl 2 and BChl 3, the backward transfer is greatly suppressed and BChl 3 is well populated. In addition, the wave-like motion induced by the quantum superposition between BChls 1 and 2 can overcome an energy barrier separating them, thereby inhibiting trapping of excitation on BChl 1. Figure 7B demonstrates that the ultrafast delocalization of excitation of BChl 6 over BChls 4, 5, 6 and 7 enables the energy flow to be unidirectional and highly efficient. Recently reported excitonic structure of baseplate with 2DES shown that baseplate states have same range energy as the nearby FMO complex [148] and predicted an alternative pathway of EET from chlorosome to RC by all the states of baseplate and FMO complexes.

### 2.4. Non-Photochemical Quenching

**Figure 8:**
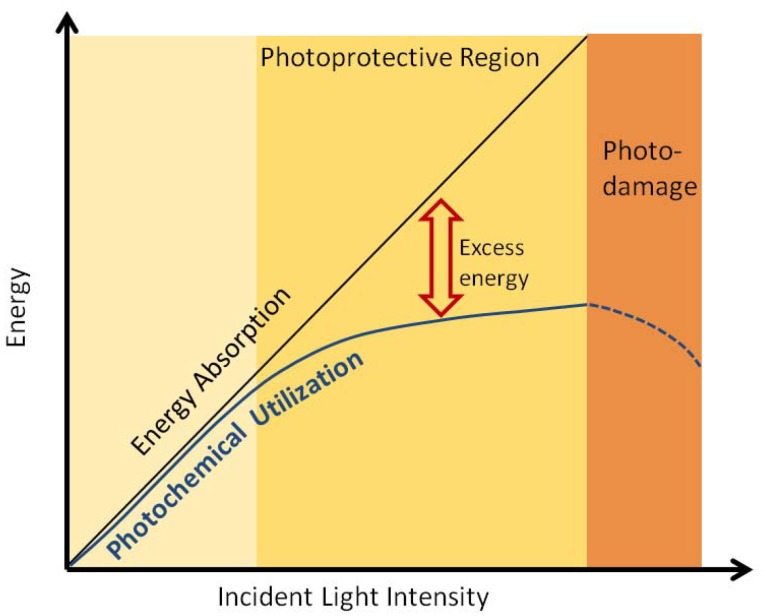
Schematic of utilization and dissipative pathways of light energy incident on the PS-II of green plants.

In a remarkable feat attained during the millennia of their evolution, photosynthetic organisms ensure optimal functionality even under disparately varying ambient conditions such as fluctuating intensity of incident radiation. Underlying the complex molecular structure and organization of the natural photosynthetic machinery is generally a clear division of light capture and chemical conversion to different functional units. This inherently demands a need for balance between the two distinct processes. Typically the functional units for light capture, *i.e*., antenna complexes, greatly outnumber the RCs, e.g., in the thylakoid membranes of chloroplasts in green plants. The chemical conversion in the RCs is the rate limiting step, particularly in the photosystem II. As a result, whenever plants are exposed to light of intensity higher than optimal, the capacity of utilization of excitation energy quickly saturates whereas the capacity for solar photon absorption remains the same, as shown schematically in Figure 8. The excess excitation energy in such a scenario can prove detrimental to plants since it leads to formation of long lived triplet states of Chl by inter-system crossing eventually giving rise to highly reactive singlet oxygen species that degrade the protein environment and damage the photosynthetic machinery [149]. To prevent this dangerous situation that, in fact, is experienced frequently by plants, evolution has developed a regulatory process known as non-photochemical quenching (NPQ) [2]. This process dissipates the excess energy in a harmless manner as heat. When the trigger for this mechanism to kick-in subsides, *i.e*., ambient light intensity returns to normal, the original energy conversion functionality of the photosynthetic apparatus gets restored quickly. Among the multiple components of such photoprotective pathway that are characterized by different timescales, the rapidly reversible energy dependent quenching process, also known as qE, is the most significant. In the following discussion, we will adopt the phrases qE and NPQ interchangeably.

Experimental and theoretical research so far has uncovered many intriguing facets of NPQ, particularly from the physiological viewpoint. However, there exists a conspicuous lack of unanimous consensus on the fundamental molecular level mechanisms [3]. Considerable evidence suggests the trigger to NPQ *in vivo* is the change in lumen pH under high light conditions. The pH gradient leads the activation of a small protein PsbS and the de-epoxidation of the carotenoid violaxanthin to zeaxanthin [150,151], which are both essential for NPQ. The former is argued to promote aggregation and/or conformational rearrangement and detachment of major LHC-II trimer complexes in the PS-II supercomplexes [152,153,154]. The viewpoint of aggregation effects is typically attributed to the strong fluorescence quenching that can be observed in aggregated LHC-II complexes after isolation from membranes [155] indicating much reduced excited state lifetime as compared to that *in vivo*. The proposed conformational change is generally believed to generate sites for NPQ by influencing the excitonic couplings between different pigments. Whether such particular quenching sites are formed at the interface of adjacent LHC-II complex or reside within a single monomeric unit, is however debated. It is also possible that additional quenching sites are formed with the involvement of minor antenna complexes.

Within the traditional view, the likely candidates for NPQ sites are believed to involve closely spaced Chl molecules that form excitonically-coupled homo- or hetero-dimers/trimers of Chl molecules or with other pigments such as carotenoids [156]. As compared to a monomeric Chl molecule, such a dimeric/trimeric conformation exhibits optical properties dependent upon conformational parameters such as relative orientation and the inter-pigment separation distance. Depending upon the configuration, the lowest excited state of such a dimer-like aggregate can be optically dark and thus might well be linked to fluorescence quenching. Chlorophyll dimers, in the so called H-conformation (cofacial orientation) are for example, known to be strong fluorescence quenchers owing to the optically dark lowest excitonic state they exhibit [157] and form the basis of the phenomenon known as concentration fluorescence quenching [158,159]. This view is contested by the propositions that the mechanisms on NPQ involve charge transfer (CT) states in homo-dimers of Chls or Chl-carotenoid hetorodimers acting as quenchers [160,161,162,163]. Rather than a unique site-dependent mechanism, given the structural complexity in LHC-II, presence of more than one mechanisms is also quite likely as has been suggested by Kruger *et al*. [164]. With NMR measurements on LHC-II systems signatures of subtle conformational changes in some of the C-atoms of Chl macrocycles were detected in the aggregated or “quenched” state [165]. Extrapolating this observation on the basis of well-known high resolution structures of LHC-II, they proposed modulation of Chl-Lutein and Chl-neoxanthin interactions during transition to quenched state, pointing to their possible role as quencher sites. Unarguable identification of peculiar sites that exhibit a high potential for NPQ to take place, thus hold promise of a greater understanding of the energy transfer/dissipation pathways in PS-II. Heading towards such goals, efforts have been devoted to explore the inter- and intra-molecular energy transfer processes in possible quenching sites explicitly at the atomistic level by using the so called non-adiabatic excited state molecular dynamics (NA-ESMD) simulations [166,167]. NA-ESMD is an accurate and efficient methodology for simulating photo-induced excitation dynamics. Our preliminary studies on chlorophyll monomers have revealed important aspects of the evolution of the excited-state wavefunction during the internal conversion of the high-energy Soret band excitation to the lowest Q${}_{y}$ state [168]. We have also found only a small subset (<5%) of the ground-state equilibrium normal modes to participate in vibrational energy redistribution. In future, we will continue to extend the application of this methodology to homo- and hetero-dimers involving chlorophylls to assess their potential as excitation quenching centers.

The entire process of NPQ is essentially multiscale, *i.e*., the trapping centers being of molecular level origins and the presence of such quenchers manifests as dissipative effects on the excitation energy transport kinetics in the entire complex network. To tackle this problem, a number of groups have formulated various models incorporating NPQ for quantitative corroborations to time-resolved fluorescence measurements. The group of van Ameronen has first developed a simple coarse-grained model [169] based on supramolecular organization of PS-II, in order to simulate excitation energy transfer and charge separation. Extending it to study NPQ via fitting the fluorescence kinetics data it was argued that the overall energy transfer efficiency [170] does not strongly depend upon the precise location of quenchers, in contrast with the traditional belief lending significance to major LHC-II complexes. Similar models for dimeric PS-II in which the EET was considered to be much faster within a given complex than that between the adjacent complexes was subsequently investigated in the Valkunas group [171]. Each of the major and minor PS-II complexes was treated as a single homogeneous unit and excitation energy transfer among them was studied while attributing the quenching ability to some specific complexes. It was proposed that the NPQ trapping center located on the major LHC-II complex yields the greatest efficiency of excitation quenching. Further studies based on this model that considered random distribution of quenchers also provided an estimate for trap density as 1 quenching center per 100 monomeric pigment-protein complexes [172]. Zaks *et al.* have developed a comprehensive mathematical model for PS-II that accounts for all the processes beginning from the transfer of the excitation energy upon photo-excitation of pigments until the terminal step of ATP synthesis to form a framework for simulating NPQ in fluctuating light conditions [173]. This highly parameterized model was successfully employed to simulate quantum yield of Chl fluorescence and predicted that the NPQ does not exert a direct influence on its trigger, *i.e*., the lumen pH. This finding has an important implication that NPQ functions purely in a regulatory role of quenching the excess energy, while leaving the photochemical processes uninfluenced.

### 2.5. Charge Separation in the Reaction Center

Natural light harvesting systems consist of RC complexes that receive the excitation energy absorbed and trigger the subsequent charge separation process. Since the first report of the three-dimensional structure of the bacterial RC from *Rhodopseudomonas viridis* by Deisenhofer and coworkers [174], the structures of the RC for many photosynthetic systems, such as photosystem I (PSI) [175,176,177], photosystem II (PSII) [178,179,180,181,182,183] and purple bacteria [184,185] have been determined. Taking the PSII RC complex as an example, it consists of ten cofactors, *i.e.*, six chlorophylls (Chls), two pheophytins (Phes) and two carotenes. Four Chls and two Phes in the center of the complex are arranged in two quasisymmetric branches, *i.e.*, D1 and D2 branches. These pigments are labeled as P${}_{D1}$, P${}_{D2}$, Chl${}_{D}1$, Chl${}_{D}2$, Phe${}_{D}1$ and Phe${}_{D}2$ with P${}_{D1}$ and P${}_{D2}$ denoting the two Chls that form a special pair. Other two Chls are located on the opposite side of the RC with a distance larger than 20 Å apart from the central special pair. It is found that the charge separation and electron transfer mainly occurs in only one active-branch- the D1 branch. In contrast, multiple pathways of charge separation in the bacteria RC from *Rhodobacter sphaeroides* have been detected by the pump-probe experiments [186] and Zinth *et al*. have also performed the pump-probe experiments to investigate the electron transfer in this RC complex [187,188] recently.

Despite the fact that the crystal structures of various RCs have been well determined, the detailed mechanism of charge separation is still under debate. Recently, various experimental techniques such as photon echo [189], femtosecond transient absorption [190] and 2DES [191,192,193] have been applied to study the charge separation and electron transfer processes in PSII RC. Numerous theoretical models have also been proposed to explain the mechanism. A multimer model [194] has been employed to calculate the energy and electron transfer dynamics of PSII RC [195] as well as various spectra, such as time-resolved pump-probe spectra, circular dichroism (CD), linear absorption (OD), linear dichroism (LD), and fluorescence (FL) spectra [196]. The asymmetric excitonic model [197] provides a realistic description by yielding site energies for each pigment via fitting various optical spectra [198]. This model assumes the initial excitation localized on Chl${}_{D1}$ [199] which is proposed to be the primary electron donor and the pigment where the charge separation occurs [194], in agreement with analysis from both the theoretical calculations [189] and photo echo and femtosecond transient absorption experiments [189,190].

Novoderezhkin and coworkers [200] applied the modified Redfield theory to fit both linear and nonlinear optical spectra of the PSII RC. In a later report by Novoderezhkin *et al*. [201] the Stark spectra were calculated and two possible disorder-controlled charge separation pathways were reported with P${}_{D1}^{-}$P${}_{D2}^{+}$ and Phe${}_{D1}^{-}$Chl${}_{D1}^{+}$ as the primary charge-separated states [201], in agreement with the transient absorption (TA) kinetics measured at 77K [202]. Further calculations of stark spectroscopy on site-directed mutants revealed that the initial charge separation states of PSII RC are three mixed exciton-CT states: (P${}_{D2}^{\delta +}$P${}_{D1}^{\delta -}$Chl${}_{D1}$)${}_{673nm}^{*}$, (Chl${}_{D1}^{\delta +}$Phe${}_{D1}^{\delta -}$)${}_{681nm}^{*}$ and (P${}_{D2}^{+}$P${}_{D1}^{-}$)${}_{684nm}^{\delta *}$ , and corresponding charge separation pathways are determined as [203]

$${\left(P_{D2}^{\delta +}P_{D1}^{\delta -}{\mathrm{Chl}}_{D1}\right)}_{673nm}^{*}\to P_{D2}^{+}P_{D1}^{-}\to P_{D1}^{+}{\mathrm{Chl}}_{D1}^{-}\to P_{D1}^{+}{\mathrm{Phe}}_{D1}^{-},$$

$${\left({\mathrm{Chl}}_{D1}^{\delta +}{\mathrm{Phe}}_{D1}^{\delta -}\right)}_{681nm}^{*}\to {\mathrm{Chl}}_{D1}^{+}{\mathrm{Phe}}_{D1}^{-}\to P_{D1}^{+}{\mathrm{Phe}}_{D1}^{-}$$

and

$${\left(P_{D2}^{+}P_{D1}^{-}\right)}_{684nm}^{\delta *}\to P_{D2}^{+}P_{D1}^{-}\to P_{D1}^{+}{\mathrm{Chl}}_{D1}^{-}\to P_{D1}^{+}{\mathrm{Phe}}_{D1}^{-}$$

respectively.

**Figure 9:**
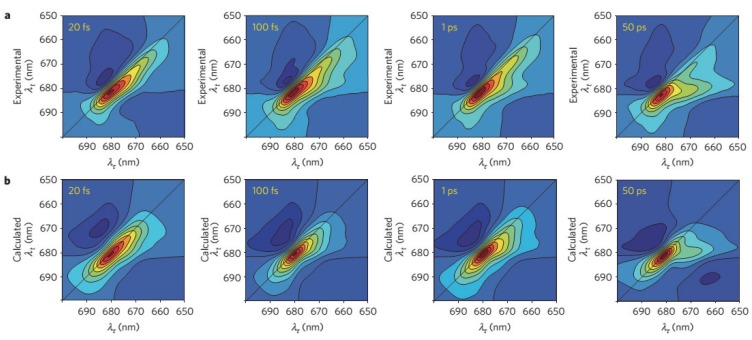
Experimental and calculated PSII RC rephasing 2D spectra at 80 K. (**a**) Experimental PSII reaction center (RC) rephasing 2D spectra; (**b**) Calculated PSII RC rephasing 2D spectra. These 2D spectra are calculated with standard Redfield theory with the disordered exciton-charge transfer (CT) model [193].

The Redfield theory has also been used to calculate the 2DES of PSII RC [193]. The calculated and experimental 2DES spectra are shown in Figure 9. Vibration assisted electronic (vibronic) coherence was detected in PSII RC by 2DES [193,204] and was claimed to drive ultrafast, efficient charge separation in this system. Lewis and coworkers [205] have used a modified Redfield theory to simulate the 2DES of PSII RC and it is found that the cross-peak features of the simulated 2D spectra are in better agreement with the experimental data for larger system-bath coupling and weaker disorder. A two-band tight-binding model was employed by Abramavicius and Mukamel [206] to calculate 2DES of PSII RC [207] considering all available electron transfer pathways. Main experimental data in time-resolved 2D optical spectra such as peak pattern, lineshapes and time traces were well reproduced with this approach.

## 3. Energy and Charge Transport in Organic Molecules for Solar Cells

### 3.1. Organic Solar Cells

Organic solar cells based on π-conjugated polymers have attracted increasing attention in recent years owing to their promising advantages in terms of low cost, versatility of functionalization, thin film flexibility, and ease of processing [208,209,210,211]. Although organic solar cells have yet to possess the photocurrent efficiencies of their inorganic counterparts (η in the range of 10%–20%), they have the potential to compete effectively with alternative solar cell technologies, quite evident from the entry of soluble light-emitting diodes based on organic semiconductors in the commercial markets [211].

**Figure 10:**
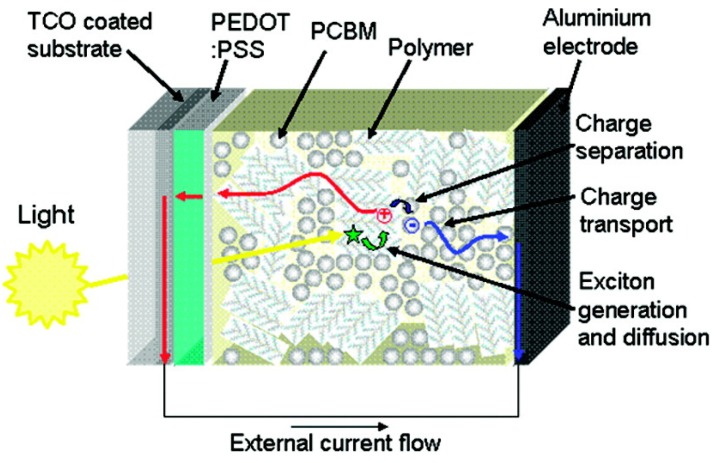
Schematic layout of the function of a typical polymer:fullerene photovoltaic devices. PCBM is [6,6]-phenyl-C61-butyric acid methyl ester, PEDOT is poly(3,4-ethylenedioxythiophene), PSS is poly(4-styrenesulfonate), and TCO is transparent conductive oxide [221].

The first generation of organic solar cells were single layer devices in which organic layers were sandwiched between two metal electrodes having different work functions [210,212]. The reported photocurrent efficiencies of these devices were generally poor due to the formation of a Schottky barrier between the p-type organic layer and the metal with lower work function [213,214]. Relatively efficient photocurrent generation in an organic device was first reported by Tang in 1986 [215], achieving a photocurrent efficiency of 1% for donor/acceptor bilayer devices fabricated from copper phthalocyanine and a perylene tetracarboxylic derivative. However, such bilayer devices often suffered from the drawback of a short exciton diffusion length which is limited by the thickness of organic layers. This results in a low quantum efficiency as the excitons need to reach the heterojunction interface prior to their decay to the ground state [216] in order for charge separation to take place. The observation of photoinduced electron transfer from the excited state of a conducting polymer onto buckminsterfullerene (C${}_{60}$) [217,218] and the enhancement of photoconductivity upon blending C${}_{60}$ in the conjugated polymers have opened up a new pathway to develop bulk heterojunction devices as the next generation of organic solar cells [219,220,221]. The schematic functional layout of such bulk heterojunction organic solar cells is shown in Figure 10. Bulk heterojunction involves a bicontinuous and interpenetrating network of donor and acceptor components in a bulk volume. This dramatically increases the interfacial area between the donor and acceptor phases, to which the excitons can easily migrate for subsequent dissociation. Much improved photocurrent efficiencies, as compared to bilayer heterojunction devices, can thus be achieved for bulk heterojunction polymer-fullerene devices [210].

The process of converting light into electric currents in an organic solar cell can be summarized in a series of steps as follows [208,209,210,211]. Absorption of a photon leads to the formation of an exciton, and this exciton subsequently migrates to the donor-acceptor interface where it may be quenched by electron transfer from donor to acceptor. However, the early part of this process does not directly lead to free electron and hole carriers, instead, it results in coulombically bound electron-hole pairs, which, under the influence of a strong local field in the interface, dissociate into free charge carriers to be transported within the organic semiconductor to respective electrodes.

The photovoltaic power conversion efficiency of an organic solar cell is determined by the number of generated charge carriers that are collected at the electrodes. The fraction of photons absorbed ($\eta_{\mathrm{abs}}$), the fraction of electron-hole pairs that are dissociated ($\eta_{\mathrm{diss}}$), and finally the fraction of (separated) charges that are collected by the electrodes ($\eta_{\mathrm{out}}$), combine to determine the overall photocurrent efficiency ($\eta_{j}$) [210]:

(5) $$\eta_{j}=\eta_{\mathrm{abs}}\times \eta_{\mathrm{diss}}\times \eta_{\mathrm{out}}$$

The fraction of photons absorbed is a function of the optical band gap, the extinction coefficient and the absorbing layer thickness. Conjugated polymers typically have large extinction coefficients, but their large band gaps do not match well with a sizeable portion of the solar spectrum, thus limiting the fraction of the absorbed incident solar light [222]. In order to enhance incident photon absorption, a tandem cell architecture in which two solar cells with different absorption characteristics are linked to absorb different parts of the solar spectrum have recently been constructed, for example by employing a transparent titanium oxide interface layer separating and connecting two cells in tandem [223]. In addition, controlling the morphology of the photoactive layer by blending conjugated polymers with electron acceptors, such as soluble fullerene derivatives, is crucial in splitting excitons into free charge carriers. The energy levels of the donor and the acceptor are also critical factors that determine the efficiency of exciton dissociation at the donor-acceptor interface. Once the charge separation occurs, the charge carriers need to travel towards respective electrodes within their lifetime, which necessitates a driving force. A gradient in the electrochemical potentials of electrons and holes is built up in the donor-acceptor interface. This gradient contributing to field-induced drift of charge carriers is determined by the free energy of the photoinduced charge-separated state and, in turn, by the difference between the highest occupied molecular orbital (HOMO) of the donor and the lowest unoccupied molecular orbital (LUMO) of the acceptor. Another driving force is attributed to the concentration gradients of the respective charge carrier species, which leads to a diffusion current. It is generally believed that thin film devices are field-drift dominated, while thick devices see more charge-carrier diffusion under concentration gradients [210].

### 3.2. Charge Transport in Organic Semiconductors

#### 3.2.1. Theoretical Models

Theoretical study of charge transport in organic molecular crystals has a long and rich history dating as back as 1950s to the pioneering work by Holstein [224,225]. A quasiparticle named the 1D Holstein polaron was introduced to account for exciton-phonon dynamics in molecular crystals. Although this theory has been widely used to qualitatively interpret experimental data, including temperature dependent band narrowing effect as well as the crossover from bandlike to hopping transport with increasing temperature, it is based on a perturbation theory and thus limited to the case of very narrow electronic bands and local (diagonal) exciton-phonon coupling. Theories based solely on the original Holstein model cannot fully describe the transport mechanisms in organic molecules, and more general models are needed to consider both local and nonlocal (off-diagonal) exciton phonon coupling. An attempt towards this direction was made by Munn and Silbey [226,227] to describe electronic coupling as well as local and nonlocal electron-phonon interactions of arbitrary strength over a wide range of temperatures. The nonlocal coupling was found to increase polaron binding energy and influence its bandwidth. In contrast to the local coupling which always narrows the bandwidth, the nonlocal coupling can introduce new minima and broadening to the band shape depending on the specific microscopic parameters [228,229,230,231,232,233,234]. Furthermore, the nonlocal coupling increases scattering and leads to a reduction of the band-like contribution to the charge transport. Based on the Holstein-Peierls-type Hamiltonian, Bobbert and coworkers presented a microscopic charge transport theory [235,236], which can reproduce the experimental electron and hole transport properties in naphthalene using microscopic parameters obtained from ab-initio calculations. However, this model only considers the coupling of electron to the optical modes and neglects the contribution of intramolecular modes. Recently, mixed quantum-classical (quantum for electrons and classical for vibration) non-adiabatic dynamics has been proposed by Hultell and Stafstrom [237] as well as Troisi and Orlandi [238] to investigate the intricate band-like and hopping charge transport mechanisms in organic semiconductors. With the vibrational modes treated classically, this theory remains valid only for the cases in which the thermal energy is larger than the average phonon frequency. It becomes clear that a complete understanding of charge transport in organic crystals inevitably requires self-consistent treatments of the Hamiltonian.

While the aforementioned theoretical treatments of charge transport in organic semiconductors do not take into account chemical and physical defects, realistic organic materials usually exhibit a highly amorphous character. It is thus highly desirable to develop a comprehensive theory of charge transport in organic crystals in the presence of static disorder. Disorder tends to trigger localization in highly ordered materials, and charge transport can be described through the hopping process whereby charges transfer from donor to acceptor. The well-known Marcus [239] expression for semi-classical electron-transfer rates is usually used to predict the charge hopping rates in organic semiconductors [240,241]. Although the Marcus theory greatly aids the understanding of the structure-function relationship in organic molecular crystals, it treats nuclear motion classically and assumes weak electronic coupling between donor and acceptor states. In organic semiconductors, environmental phonons include both intramolecular and intermolecular vibration modes, which often posses high frequency characteristics. Thus, more general theories beyond the semiclassical treatment for the environment need to be developed in order to correctly describe the quantum mechanical aspects of the charge transfer process [242]. Furthermore, when the electronic coupling between donor and acceptor states is strong, the Marcus theory always predicts the rates to increase with the square of electronic coupling due to its perturbation approximation, in stark contradiction with the adiabatic suppression effect with diminishing rates when the electronic coupling is large enough [243]. In order to overcome the shortcomings of the Marcus theory, several approaches beyond the perturbative and semiclassical approximation have been developed [244,245,246,247]. We next proceed to outline the main characteristics of the two models for explaining the charge transport mechanisms in organic semiconductors, *i.e*., the polaron and disorder models [248]:

**Polaron models:**  Ultra-pure organic single crystals devoid of any chemical or physical defects are the prototypical systems for studying charge transport in organic semiconductors. In such idealized systems, the charge transport depends on a subtle interplay between electronic and electron-phonon interactions. Most theoretical studies incorporating simultaneously the local and non-local electron-phonon coupling [226,227,228,229] make use of the generalized Holstein Hamiltonian given by:

(6) $$\begin{array}{ccc} H & = & \sum_{n}\epsilon_{n}a_{n}^{†}a_{n}+\sum_{n\neq m}J_{nm}a_{n}^{†}a_{m}+\sum_{q}\omega_{q}\left(b_{q}^{†}b_{q}+1/2\right)\\ & & +\sum_{nq}g_{nq}\hbar \omega_{q}\left(b_{q}+b_{-q}^{†}\right)a_{n}^{†}a_{n}+\sum_{n\neq m,q}f_{nm,q}\hbar \omega_{q}\left(b_{q}+b_{-q}^{†}\right)a_{n}^{†}a_{m} \end{array}$$

where $a_{n}^{†}\left(a_{n}\right)$ is the creation (annihilation) operator of an excitation (*i.e*., an exciton or a charge carrier) with on-site energy $\epsilon_{n}$, and $b_{q}^{†}\left(b_{q}\right)$ is the creation (annihilation) operator of a phonon with frequency $\omega_{q}$ and a wave-vector *q*. $J_{nm}$ is the electronic transfer integral coupling two molecules *n* and *m*. The electron-phonon coupling can be decomposed into local (Holstein-type) and non-local (Peierls-type) coupling arising from overall modulations of the site energy and the transfer integral, respectively. In Equation (6), the $g_{nq}$ and $f_{nm,q}$ terms denote the local and nonlocal electron-phonon coupling constants. In organic crystals, consideration of both of these coupling terms is vital [248].

**Figure 11:**
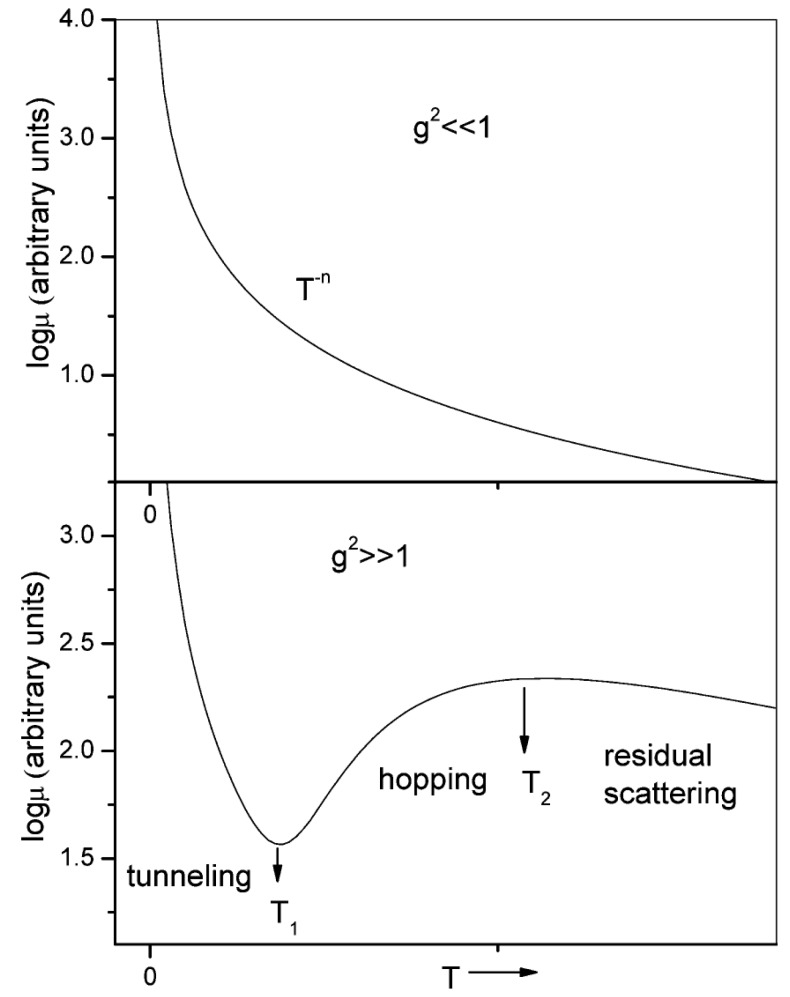
Mobility as a function of temperature for weak (top panel) and strong (bottom panel) electron-phonon coupling predicted by Holstein polaron model [248].

According to the general microscopic models [225,248,249], the mobility is mainly determined by two mechanisms:

(7) $$\mu =\mu_{\mathrm{tun}}+\mu_{\mathrm{hop}}$$

where $\mu_{\mathrm{tun}}$ represents band-like transport (coherent charge transfer) that dominates at low temperatures, and $\mu_{\mathrm{hop}}$ is related to hopping transport that mainly takes place in the high temperature regime. Figure 11 shows temperature dependence of mobilities in the weak and strong electron-phonon coupling regimes as predicted by the Holstein model. In the case of weak coupling ($g^{2}\ll 1$), the charge transport is dominated by band-like transport mechanism ($\mu ∼T^{-n},n>0$) across the entire temperature range. For intermediate coupling ($g^{2}\leq 1$), mobility exhibits band-like behavior at low temperatures, while temperature dependence of mobility becomes much weaker at high temperatures [250]. For strong coupling ($g^{2}\gg 1$), three distinct regimes appear showing band-like behavior for the low temperature ($T<T_{1}$) regime, hopping mechanism at the high temperature regime, and a crossover from band-like transport to hopping transport in the intermediate regime. When temperature reaches a very high value ($T=T_{2}$) at which the thermal energy itself can dissociate the polaron, the residual scattering effects come into picture causing lowering of the mobility.

In order to correctly describe the mobility in the finite-temperature regime, a variational method was proposed combining Merrifield’s transformation with Bogoliubov’s theorem for 1D Holstein models [251]. This method can characterize the crossover from band-like transport to hopping transport in molecular crystals, and the calculated charge-carrier mobilities were found to compare well with experiments on ultrapure naphthalene crystals across the whole experimental temperature range. However, Merrifield’s transformation is applicable only in the small polaron regime, and the non-local electron-phonon coupling was not included in this approach. More recently, a theory based on nonperturbative evaluation of the Kubo formula for the carrier mobility [252,253] has been put forth, showing several improvements including the elimination of low temperature singularity that often appears in theories based on narrow-band approximations and the temperature dependence and anisotropy of carrier mobility. Following up the Munn-Silbey transformation method, Zhao and coworkers [228,254] devised a self-consistent routine to determine the optimal transformation coefficients. Improved results have been obtained on the temperature dependence of transport properties in an extended Holstein model incorporating both local and non-local electron-phonon coupling.

**Disorder models:**  The chemical and physical impurities found commonly in organic materials make it imperative that the description of charge transport in organic crystals must take into account the effect of disorder. As disorder tends to inhibit band-like charge transport, it can then be described by hopping mechanism with charge carrier migrating between interacting molecules. For amorphous conjugated polymer films, the disorder can be classified into diagonal and off-diagonal types with the former being induced both by electrostatic effects and a distribution of the effective conjugation length while the latter arising from the relative separation and orientation between neighboring molecules [248].

In theory, random walk schemes combining the technique of kinetic Monte-Carlo (KMC) simulation are usually applied to treat charge transport in disordered organic materials [255]. The general steps in a typical KMC simulation are as follows [255]. First, a molecule within the bulk is arbitrarily chosen as the starting site for the charge, from which charge migration is only allowed to its nearest neighboring sites. The charge transfer rates to all the neighboring sites are then computed using parameters obtained via ab-initio calculations. Next, the probability of hopping to the αth neighbor is evaluated as $p_{\alpha }=k_{\alpha }/\sum_{\beta }k_{\beta }$, where $k_{\alpha }$ is the charge transfer rate. In order to determine the next site the charge will hop to, a random number *r* in the uniform distribution U(0,1) is generated. Hopping to αth neighboring site takes place if the expression $\sum_{\beta =1}^{\alpha -1}p_{\beta }<r<\sum_{\beta =1}^{\alpha }p_{\beta }$ is satisfied. The simulation continues until the diffusion distance exceeds the intermolecular spacing by 2–3 orders of magnitude. A sufficiently large number of independent KMC simulations must be performed in order to achieve reliable ensemble averages so as to obtain a linear relationship between the mean-square displacement and the simulation time upon which the diffusion coefficient can be computed. From the resulting diffusion coefficient, the mobility is finally obtained by the Einstein formula $\mu =eD/k_{B}T$, where *e* is the electron charge, *D* is the charge diffusion coefficient, and $k_{B}$ and *T* are Boltzmann constant and temperature, respectively.

In the above random walk simulations, charge transfer rate is the most essential input parameter. The widely used charge transfer rate based on the semi-classical Marcus theory [239] can be written as:

(8) $$k=\frac{V^{2}}{\hbar^{2}}\sqrt{\frac{\pi }{\lambda k_{B}T}}\exp\left(-\frac{{\left(\lambda +\Delta G^{0}\right)}^{2}}{4\lambda k_{B}T}\right)$$

where *V* is the transfer integral between the initial state (donor molecule) and final state (acceptor molecule), λ denotes the reorganization energy induced by geometry relaxation during the charge transfer, and $\Delta G^{0}$ is the variation of the Gibbs free energy during the charge transfer process. It is noted that the Marcus theory is based on a first-order perturbative treatment of the transfer integral and applicable only in the high temperature limit. When the charge is strongly coupled with high frequency intramolecular vibration modes, quantum mechanical effects will assume dominant roles in the charge transfer process. In addition, the transfer integrals in organic crystals usually vary by several orders of magnitude [256,257], invalidating the perturbation theory.

An alternative quantum mechanically derived multi-mode formula for the charge transfer rate under the displaced harmonic oscillator approximation can be obtained from the Fermi Golden Rule (FGR) as [255]:

(9) $$\begin{array}{ccc} k & = & \frac{V^{2}}{\hbar^{2}}\int_{-\infty }^{\infty }dt\times \exp\{i\Delta G^{0}/\hbar -\\ & & \sum_{j}S_{j}\left[\left(2n_{j}+1\right)-n_{j}e^{-i\omega_{j}t}-\left(n_{j}+1\right)e^{i\omega_{j}t}\right]\} \end{array}$$

where $S_{j}$ is the Huang-Rhys factor characterizing the coupling strength between charge carrier and *j*th phonon mode, and $n_{j}=1/\left(e^{\hbar \omega_{j}/k_{B}T}-1\right)$ is the occupation number for *j*th phonon mode with frequency $\omega_{j}$. Expanding the exponential factors in Equation (9) and evaluating the integral analytically leads to the Bixon-Jortner formula for the charge transfer rate [258]. In the high temperature limit, Equation (9) recovers the Marcus formula (Equation (8)). Although the FGR incorporates the nuclear tunneling effect, it is still based on a perturbative approximation. The generalized nonadiabatic transition state theory (GNTST) [259] based on the Zhu-Nakamura nonadiabatic transition probability [260] provides a powerful tool to evaluate the charge transfer rates from weak to strong coupling. Simulation results show that the GNTST-calculated hole mobility of oligothiophenes single crystal is three times as large as that from the semiclassical Marcus theory, which can be attributed to the quantum nuclear tunneling and nonperturbative effects [259].

#### 3.2.2. Charge Transport in Organic Molecules

Once the photo-generated excitons dissociate into free charge carriers (electrons and holes), they are transported to the opposite electrodes with an efficiency that depends critically on their mobilities. The charge transport properties in organic molecules strongly depend upon factors such as intermolecular electronic couplings, electron-phonon interactions and structural defects. As a result, the measured charge-carrier mobilities can vary over several orders of magnitudes as a function of sample quality.

**Figure 12:**
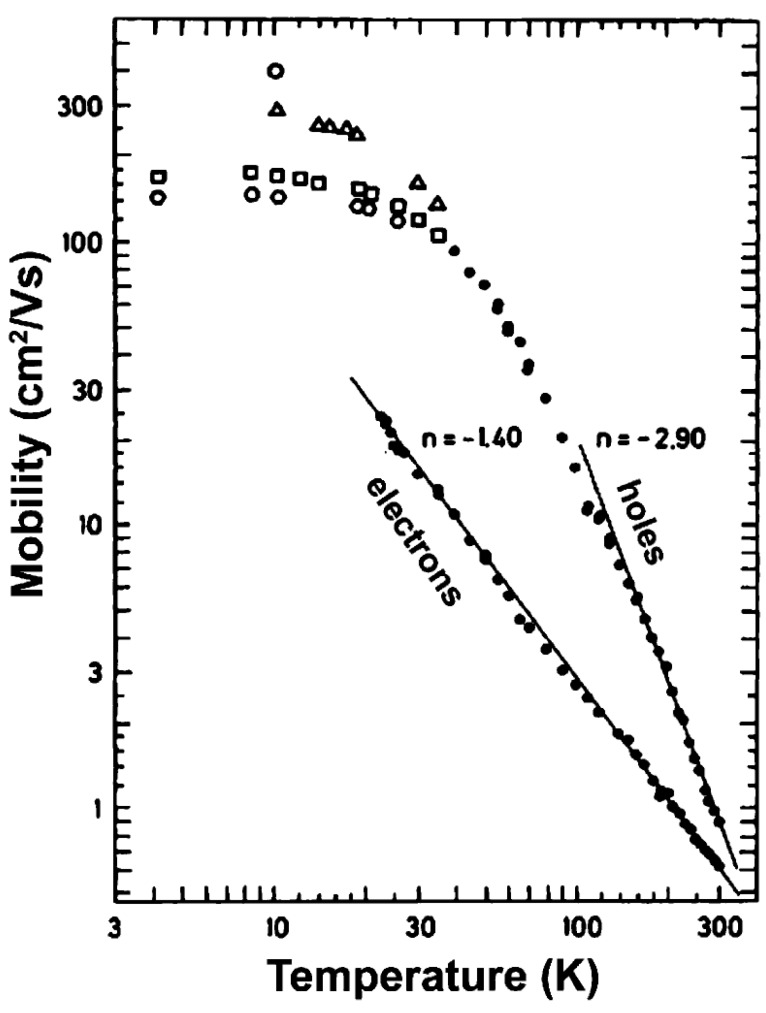
Electron and hole mobilities in ultrapure naphthalene as a function of temperature. The applied electric field is approximately parallel to the crystallographic a direction [261].

While significant efforts have been devoted to the molecular level understanding of factors affecting the charge carrier mobilities in organic semiconductors, the design of high mobility organic materials is still driven by empirical experiments which is partly due to the lack of a unified theoretical treatment of charge transport in organic semiconductors. In ultra-pure organic single crystals, charge transport at low temperature can be described in the framework of band transport mechanisms [261]. The transfer integral and electron-phonon coupling are two key parameters that determine the charge transport mechanisms (band or hopping transport) in organic semiconductors. The transfer integral is directly related to the bandwidth, and the electron-phonon coupling can alter electronic band structures. As the temperature is lowered, the electron-phonon coupling is decreased, and correspondingly, the mobility increases. Hence, the temperature dependence of charge carrier mobility ($\mu \propto T^{-n}$, *n* typically varies between 0.5 and 3) can be used to test whether band transport occurs in organic semiconductors. Figure 12 shows the electron and hole mobilities in ultrapure naphthalene as a function of temperature. The decrease in mobilities with temperature for electron and hole clearly indicates the band-like transport. Although the majority of the experimental observations [262,263,264] indicate negative temperature dependence of mobility in the 100–300 K range, it is argued that the band and delocalized picture are inconsistent with the analysis of experimental data which reveals a mean free path of charge carriers of the same order of magnitude of the unit cell at room temperature [265]. Troisi *et al.* recently proposed that the charge transport mechanism in organic semiconductors at room temperature is neither band-like nor a combination of thermally activated hopping and band-like [238]. Computational investigations show that for many organic semiconductors, the fluctuation amplitudes of the transfer integral are comparable to the transfer integral itself. Under this circumstance, the translational symmetry of the electronic Hamiltonian is greatly destroyed and the band-like transport picture is invalid. The transfer integral fluctuation is mainly attributed to the low frequency modes, and it is these low frequency intermolecular vibrations that dynamically localize the charge carriers. As localization is mainly originated from the dynamic disorder in the transfer integral, decreasing the temperature will attenuate dynamic disorder, and correspondingly, the mobility will increase. The proposed model can successfully explain the spectroscopic observation of localized carriers as well as band-like charge transport, and the authors suggest that the most efficient way to improve the charge mobility in organic semiconductors is to reduce the thermal electronic disorder.

**Figure 13:**
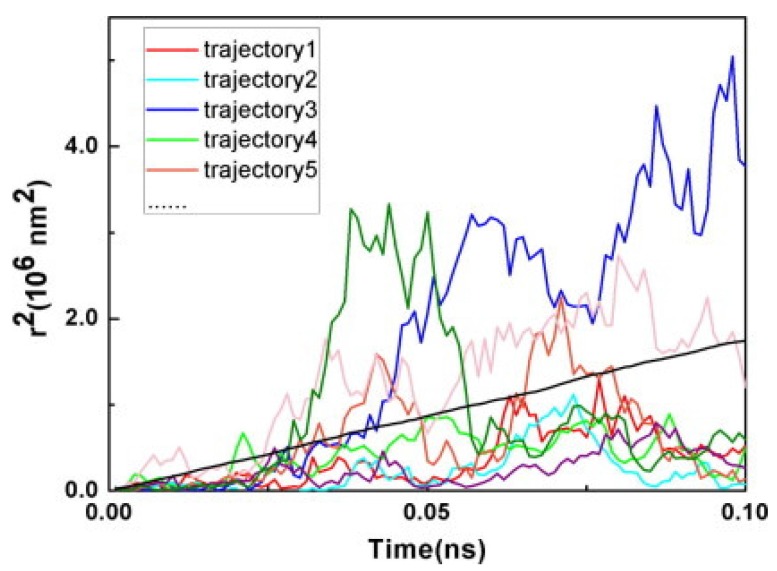
Square displacement of individual trajectory versus the transport time. The straight line is the average values of the square displacements versus time [272].

As is evident from the ongoing discussion, single crystals of organic semiconductors provide ideal test-beds to study fundamental charge transport physics in the cleanest possible systems. However, there usually exist impurities and defects in many organic materials since they present a highly amorphous character, and the theoretical descriptions of charge transport in such organic disordered materials are mainly based on hopping model in the framework of Monte Carlo method as described above. There has occurred a tremendous progress in the theoretical investigations on the molecular design from the perspective of charge transport in organic molecules [240,248,255,266]. According to Equations (8) and (9), the reorganization energy and the intermolecular transfer integrals are two key parameters that control the charge transport properties in organic semiconductors, such that a small value of the former and a large one of the latter can speed up the charge transfer process. There are various quantum chemistry based methods available to calculate the transfer integral, such as the energy splitting in dimer method [267], the site energy correction method [268] and the direct coupling methods [269]. The reorganization energy is usually calculated by the normal mode analysis method [270] and the four-point method based on the adiabatic potential energy surface method [271]. The obtained parameters are then used as inputs to Equation (8) or (9), with which the charge transfer rate between all neighboring molecules can be obtained, and charge mobility be evaluated via the Monte-Carlo method. The application of these theoretical methods to organic semiconductors can provide useful design principles for organic materials with high charge mobility. Lin *et al.* recently investigated the charge transport properties in four perylene diimides based n-type organic semiconductors by employing a tunneling enabled hopping model [272]. It is found that the substitutions at the bay positions of the perelene core can significantly affect the charge transport properties through the modification in molecular conformation as well as the stacking networks in the crystals. Using the calculated intermolecular transfer integral and reorganization energy parameters, KMC method was applied to simulate the charge transfer properties. The squared displacement of each trajectory versus the transport time is shown in Figure 13 and the diffusion coefficient can be obtained as the slope of the straight line which is the average result of many trajectories. The authors also found that all the four organic semiconductors under their investigation presented “band-like” temperature dependence of mobilities. This, however, is in surprising contradiction with the localized charge hopping model used in their simulation. Based on the fact that the electron is strongly coupled to the high frequency vibration modes, the authors ascribed this behavior to purely nuclear tunneling effect for localized charges. The “tunneling enabled hopping” model proposed can explain the paradoxical experimental observations leading sometimes to a delocalized band-like transport and sometimes to the existence of localized charge carriers [273].

### 3.3. Photoinduced Charge Separation in Organic Solar Cells

**Figure 14:**
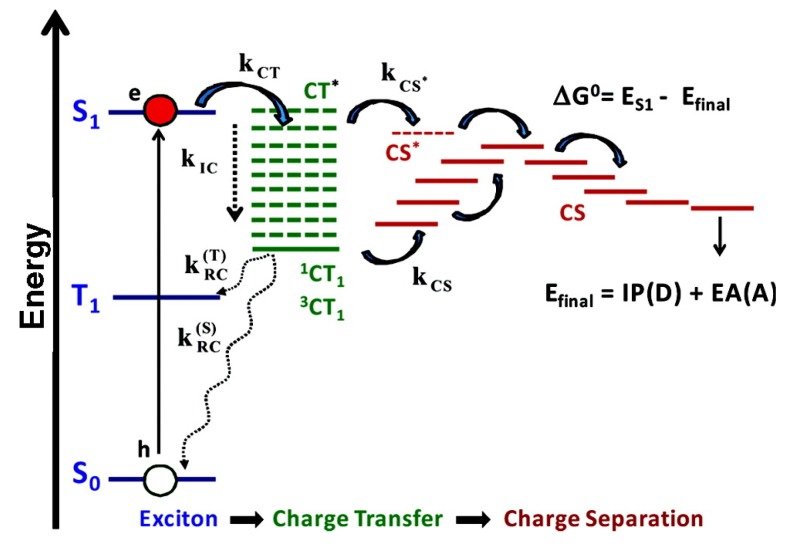
Energy level diagram describing the main processes involved in the photo-induced charge-carrier formation mechanism in an organic solar cell. $S_{0}$: Ground state of the donor or the acceptor, and $S_{1}$ ($T_{1}$) denote the first singlet (triplet) excited state. $k_{\mathrm{CT}}$: Exciton dissociation to form the hot charge transfer (${\mathrm{CT}}^{*}$) states. $k_{\mathrm{IC}}$: Thermal relaxation of the ${\mathrm{CT}}^{*}$ state to lowest energy charge transfer state (${}^{1}{\mathrm{CT}}_{1}$ and ${}^{3}{\mathrm{CT}}_{1}$). $k_{\mathrm{RC}}^{\left(T\right)}$($k_{\mathrm{RC}}^{\left(S\right)}$): Geminate recombination of the ${}^{3}\mathrm{CT}$ (${}^{1}\mathrm{CT}$) state to the triplet excited state (ground state). $k_{\mathrm{CS}}^{*}$: Dissociation of the hot *CT* state into a fully charge separated (*CS*) state. $k_{\mathrm{CS}}$: Dissociation of the thermally relaxed *CT* state into the *CS* state. $\Delta G^{0}$ is the Gibbis free energy change of charge separation process. $E_{\mathrm{final}}$ is the final energy of fully *CS* state, where *IP*(D) and *EA*(A) are the electron donor ionization potential and the electron acceptor electron affinity, respectively [209].

Crucially important for the efficient photocurrent generation in OPVs is the separation of photoinduced electron-hole pairs (excitons) into free electrons and holes with a high quantum yield and minimal energy loss at donor/acceptor interfaces. Contrary to the conventional inorganic photovoltaic cells such as those based on silicon p-n junctions, organic solar cells typically exhibit small dielectric constants and highly localized electronic states, which lead to strong Coulomb interaction between electron-hole pairs [274]. The exact mechanism by which organic solar cells can overcome the Coulomb attraction of the photogenerated electron-hole pair leading to separated charges is still quite far from being understood at the molecular level. The charge separation process is usually described as a two-step process [209,275,276,277,278,279]. Figure 14 schematically depicts the energy level diagram describing the main processes involved in the photo-induced charge-carrier formation mechanism in an organic solar cell [209]. Initially, the photons absorbed excite the donor molecules into $S_{1}$ singlet excited states (singlet excitons). These singlet excitons at the donor/acceptor interface can be quenched by electron transfer from donor to acceptor, generating charge transfer (*CT*) states where the electrons at acceptor and holes at donor are bounded by strong Coulomb attraction. There is then a kinetic competition between full charge separation to form dissociated charge carriers via a manifold of charge separated (*CS*) states, and loss of the energy through thermal relaxation process whereby the *CT* states relax to the lowest energy level (${}^{1}{\mathrm{CT}}_{1}$ and ${}^{3}{\mathrm{CT}}_{1}$). In addition, the charge separation process can be inhibited by the geminate recombination of a *CT* state back to either the ground state, $S_{0}$, or a triplet exciton, $T_{1}$, depending on their spin state. $\Delta G^{0}$ is the free energy change during this overall charge separation process, and a larger $\Delta G^{0}$ will result in an increased probability of escape of an electron from the Coulomb attraction of the electron-hole pairs at donor/acceptor interface.

The rates of internal conversion (*IC*) and charge separation (*CS*) processes ($k_{\mathrm{IC}}$ and $k_{\mathrm{CS}}$, respectively) determine the photo-induced charge separation efficiency. For the case of $k_{\mathrm{IC}}\gg k_{\mathrm{CS}}$, the “hot” CT state (${\mathrm{CT}}^{*}$) undergoes a fast relaxation to the lowest CT states (${}^{1}{\mathrm{CT}}_{1}$ or ${}^{3}{\mathrm{CT}}_{1}$) where the electrons at acceptor and holes at donor are still strongly Coulombically bound. The electrons and holes have to surmount the Coulomb barrier to form dissociated charge carriers, thus the exciton dissociation via this pathway is likely not an efficient process. In the opposite case, *i.e*., $k_{\mathrm{CS}}\gg k_{IC}$, hot CT excitons(${\mathrm{CT}}^{*}$) dissociate into the free charge carriers (CS state in Figure 14) [280,281,282]. This process is similar to the Onsager model for autoionization in solution [211], which states that efficient charge separation take place only if the electrons can escape the Coulomb attraction of holes before their thermal relaxation. Jailaubekov *et al.* recently applied femtosecond nonlinear optical spectroscopies and non-adiabatic mixed quantum mechanics/molocular mechanics simulation to present the real-time dynamics of hot CT exciton formation and relaxation in the phthalocyanine-fullerene OPV system [281]. The results showed that the formation of hot CT excitons and ensuing thermal relaxation to CT states at lower energies with shorter electron-hole distances are on the timescale of $10^{-13}$ s and $10^{-12}$ s, respectively. They argued that it is this time limit for the competitive charge separation channels that leads to the efficient photocurrent generation. Grancini *et al.* performed the transient absorption spectroscopic studies on the excited state evolution in a PCPDTBT/PC${}_{60}$BM heterojunction [282], and found that exciton dissociation occurs within the first 50 fs, creating both the interfacial CT states and the polaron species. They attributed the enhancement of charge dissociation to the higher degree of delocalization of the hot CT states with respect to the relaxed ones. These findings indicate that the charge carriers could undergo a few ultrafast jumps after the CT process in order to reduce the probability of hot CT states relaxation and thus get efficiently separated. However, Vandewal *et al.* challenge this viewpoint by performing a detailed analysis of electroluminescence emission spectra and external quantum efficiency spectra for a number of D/A interfaces [283]. To their surprise, the field dependence and absolute value of the quantum yield have nothing to do with whether or not the D, A or CT states with an energy higher than that of $CT_{1}$ are excited. They claimed that the present state of the art bulk heterojunction layers produce a quantum efficiency higher than 90% due to efficient dissociation of $CT_{1}$ into free charge carriers instead of the higher energy “hot” states.

## 4. Outlook

A growing body of studies addressing the connection between coherent excitation dynamics and energy transfer efficiency has inspired numerous discussions on whether quantum mechanical effects play significant roles in energy transfer processes in biological systems. Through millennia of evolution, nature seems to have developed a variety of tools that enable biological systems to operate in optimal regimes in which electronic coupling within the system and its coupling to the environment are comparable so that both contributions do not merely coexist but cooperate in an elegant manner. However, the fact that the experimental setup are different from the natural sunlight condition raises two intriguing questions. On one hand, the coherent dynamics observed in experiments on light harvesting, using ultrafast coherent laser pulses, has invigorated the question to what extent this effect is present under natural light harvesting sunlight-continuous incoherent illumination conditions [284,285,286]. Along this direction, two-dimensional spectroscopy with incoherent light as proposed by Turner *et al.* [287] can provide a useful tool to clarify whether coherent dynamics resulting from artificial excitation processes reflects the characteristics of biological systems under natural incoherent excitation conditions. On the other hand, conventional femtosecond optical spectroscopy deals with a large number of transients coming from individual molecules and are thus subject to inhomogeneous broadening and dephasing that may obscure certain dynamics features. The recent single molecule experiments pioneered by van Hulst and co-workers [288,289] permit real-time monitoring of not only electronic populations and vibrational wavepackets, but also of electronic coherences in individual molecules.

Construction of predictive models that can correlate material properties to photovoltaic device efficiency is a crucial step in the optimization of organic solar cells. Such models can provide guiding principles on materials and device design, thus facilitate the improvement of device performance. Although the semiclassical Onsanger-Braun theory is widely applied to calculate the exciton dissociation rate for a variety of organic photovoltaic devices, the influence of phonons is often neglected. Recently, Yao, Yang and Zhao studied exciton dissociation under the influence of a phonon bath by using the HEOM approach within the Wigner function formalism [290]. Significant deviations from the Onsager-Braun theory were found, demonstrating phonon-induced quantum effects. It remains a challenging task to model charge transport phenomena in a quantitatively accurate manner. Progress has been made in the development of universal theories that can explain paradoxical experimental observations leading sometimes to a band-like transport and sometimes to localized charge carriers, yet a significant ground remains to be covered to reach a satisfactory understanding of the underlying phenomena.
